# SGLT2is and Renal Protection: From Biological Mechanisms to Real-World Clinical Benefits

**DOI:** 10.3390/ijms22094441

**Published:** 2021-04-23

**Authors:** Giovanna Leoncini, Elisa Russo, Elisabetta Bussalino, Cecilia Barnini, Francesca Viazzi, Roberto Pontremoli

**Affiliations:** Department of Internal Medicine, the University of Genoa and IRCCS Ospedale Policlinico San Martino, 16132 Genova, Italy; giovanna.leoncini@unige.it (G.L.); elisa24russo@gmail.com (E.R.); betta.bussalino@gmail.com (E.B.); cecilia.barnini@gmail.com (C.B.); francesca.viazzi@unige.it (F.V.)

**Keywords:** SGLT2 inhibitors, kidney protection, kidney disease

## Abstract

In recent years, following the publication of results from several RCTs, first on cardiovascular and more recently on renal outcomes, SGLT2is have become the standard of care to prevent diabetic kidney disease and slow its progression. This narrative review focuses on biological mechanisms, both renal and extrarenal, underlying kidney protection with SGLT2is. Furthermore, data from cardiovascular as well as renal outcome trials, mostly conducted in diabetic patients, are presented and discussed to provide an overview of current uses as well as the future therapeutic potential of these drugs.

## 1. Introduction

Over the last two decades, at variance with cardiovascular (CV) morbidity and mortality, which has progressively declined in patients with diabetes over the last two decades, the trend in microvascular complications has remained substantially unchanged [1]. In particular, diabetic kidney disease (DKD) accounts for up to 60% of new patients beginning renal replacement therapy (RRT) worldwide and its prevalence has shown only a slight reduction in recent years [2].

About a decade ago, following concerns regarding the CV safety of some newly developed antihyperglycemic drugs, regulatory authorities in the U.S., and later in Europe, required that new glucose-lowering drugs be tested to prove their CV safety before allowing them into the market [3]. Since then, a large number of trials have shown some new classes of anti-diabetic drugs (namely sodium glucose cotransport 2 inhibitors (SGLT2is) and glucagon-like peptide 1 agonists (GLP1a)) to not only be safe and effective, but also be capable of providing additional CV and renal benefits beyond their glucose-lowering effect.

For several decades, according to the traditional glucocentric vision, long-term diabetic complications were merely considered to be a consequence of hyperglycemia and therefore deemed preventable by rigorous glycemic control. Unfortunately, this therapeutic approach provided only modest results in terms of preventing micro- and macrovascular complications. More recently, and due in part to the results of the latest intervention trials with newly developed drugs, the paradigm for diabetes management has shifted toward a multi-factorial, organ-protection-centered approach. However, patients with diabetes remain at high risk of developing CV and renal complications despite the achievement of glycemic, blood pressure (BP) [4], and lipid targets, and the use of renin–angiotensin–aldosterone (RAAS) inhibitors. Therefore, there is a great need to identify new therapeutic strategies to optimize cardiorenal protection. This narrative review focuses on the biological action mechanisms of SGLT2is and reviews available data from RCTs that support the outstanding renal-protective effect of these drugs.

## 2. Mechanisms of Renal Protection with SGLT2

**Figure 1:**
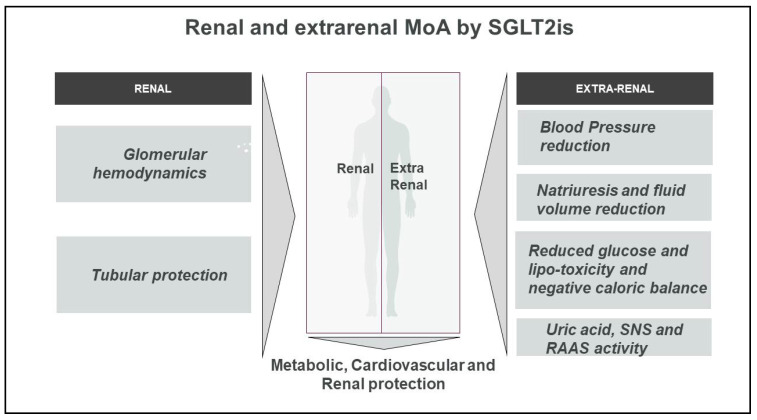
Renal and extrarenal mechanisms of action by SGLT2is. Selective inhibition of SGLT2 in proximal renal tubule prevents glucose reabsorption and entails several potentially favorable effects. Glycosuria concurs to euglycemia, lowers HbA1c, and reduces glucotoxicity, preserving beta cell function. As for extra-glycemic effects, SGLT2is promote diuresis and natriuresis and determine a mild reduction in extracellular fluid, especially interstitial fluid. Effective blood volume and blood pressure reduction ensues. Abbreviations: MoA, mechanisms of action; RAAS, renin angiotensin aldosterone system; SNS, sympathetic nervous system.

**Figure 2:**
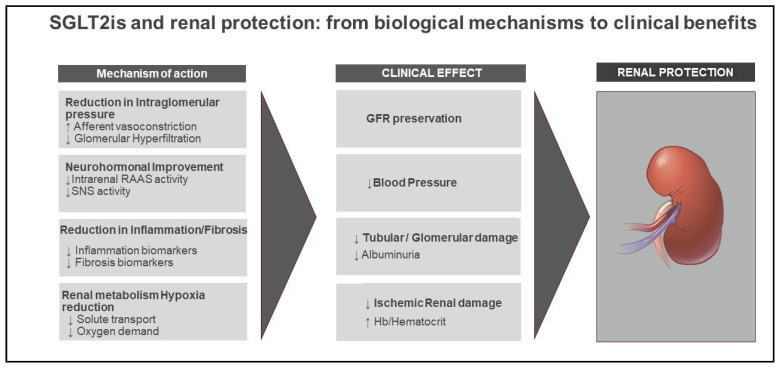
SGLT2is and renal protection: from biological mechanisms to clinical benefits. Renal protection by SGLT2 inhibitors is likely multifactorial. In the short term, SGLT2is promote diuresis and natriuresis as well as tubuloglomerular feedback activation, resulting in afferent arteriole vasoconstriction and a reduction in intraglomerular pressure, filtration fraction, and eGFR. Over a longer term, SGLT2is induce a reduction in inflammatory as well as interstitial fibrosis biomarkers together with an increase in hematocrit and mitigation of hypoxia in tubular cells. These changes result in a reduction in blood pressure and urine excretion and GFR preservation over time.

Glycosuric agents have been shown to exert their favorable action through several renal as well as extrarenal mechanisms (Figure 1 and Figure 2).

### 2.1. Renal Mechanisms

#### 2.1.1. Glomerular Hemodynamics

By inhibiting sodium absorption in the proximal tubule, SGLT2is increase distal delivery of sodium chloride to the macula densa, activating tubulo-glomerular feedback and ameliorating glomerular hypertension and hyperfiltration through reversal of both afferent arteriole vasodilation and efferent arteriole vasoconstriction typically observed in diabetes [5,6,7]. Moreover, NaCl delivery to the distal nephron reduces the glomerular filtration rate (GFR) by increasing hydrostatic pressure in Bowman’s space [6]. Natriuresis may further be promoted through the suppression of sodium–hydrogen exchanger (NHE3) activity, usually upregulated in diabetes [8].

#### 2.1.2. Tubular Protection

Low-grade chronic inflammation is associated with DKD, mainly due to increased oxidative stress and activation of pro-inflammatory pathways that may promote disease progression [9,10]. Furthermore, chronic hypoxia, hyperglycemia, and RAAS activation, via TGFβ and connective tissue growth factor (CTGF), contribute to fibrogenesis [7]. Several studies demonstrated that treatment with SGLT2is reduces markers of inflammation and fibrosis in proximal tubular cells [11] and in animal models [12,13,14]. Furthermore, the anti-fibrotic action of SGLT2is seems to be mediated by mTORC1 inhibition [15]. By decreasing sodium and glucose tubular load, SGLT2is improve tissue oxygenation, thus reducing the production of hypoxia-inducible factor- 1α (HIF-1α) [16] and providing tubular protection. Calories lost through glycosuria may activate a starvation signaling pathway, leading to gluconeogenesis, fatty acid oxidation, and ketogenesis [17]. Furthermore, SGLT2is induce upregulation of adenosine-monophosphate-activated protein kinase (AMPK), a cellular fuel gauge [18], and sirtuin-1 (SIRT1), metabolism and stress response regulator [19], which in turn increase FGF21/PGC-1α axes activity, leading to restoration of impaired autophagic flux, reduction in inflammation, and eventually decreased cellular stress. SIRT-1 is also responsible for HIF-2α activation with consequent enhanced erythropoietin production [17].

### 2.2. Extrarenal Mechanisms

#### 2.2.1. Blood Pressure Reduction

**Figure 3:**
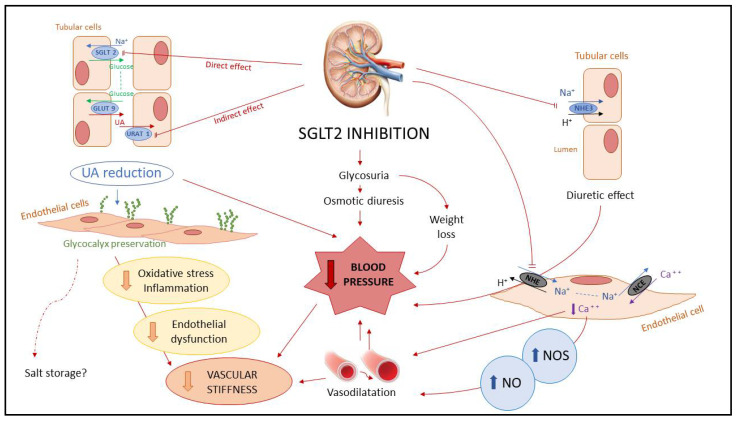
Proposed systemic renal-protective pathways with SGLT2 inhibitors. The potential pathways linking SGLT2is with blood pressure reduction and vascular stiffness improvement involve the intravascular volume depletion via osmotic diuresis due to glycosuria, the lowering of blood glucose (and glucotoxicity), blood serum uric acid levels, and body mass. SGLT2is increase glucose concentrations in the proximal tubules, wherein glucose competes with urates for the transporter GLUT9, reducing urate reabsorption and then increasing renal uric acid excretion as a direct effect. This was speculated to be an indirect effect of SGLT2is on URAT1, as a consequence of the glycemia reduction on insulin amount and other mechanisms not insulin-mediated. It was demonstrated that restoration of the integrity of the endothelial glycocalyx in human cells, possibly resulting in atheroprotective effect and contrasting endothelial dysfunction mediated by oxidative stress and inflammation, leads to a reduction in arterial stiffness. An emerging hypothesis states that sustaining salt storage in the glycocalyx contributes to developing hypertension due to impaired excretory ability of sodium at the kidney level. Furthermore, SGLT2is inhibit NHE in tubular cells, acting as a proximal diuretic; and also inhibit endothelial NHE, leading to decreased intracellular calcium, increasing endothelial NOS and NO levels, allowing vasodilatation. Abbreviations: GLUT, glucose transporter; NHE, Na+/H+ exchanger; NOS, nitrite oxide synthase; NO, nitrite oxide; SGLT2is; sodium-glucose cotransporter 2 inhibitors; URAT, urate transporter.

Recent metanalyses showed an association between the SGLT2is treatment and sustained lowering of systolic and diastolic BP by 4–6 mmHg [20] and 1–2 mmHg [21], respectively. Interestingly, empagliflozin reduced systolic BP both in dipper and non-dipper patients with type 2 diabetes [22], whereas the effects of SGLT2is on long-term and short-term BP variability have not yet been established. SGLT2is may produce BP changes by a number of molecular mechanisms, mainly by inducing intravascular volume depletion via glycosuria and osmotic diuresis. Nonetheless, the antihypertensive effect of SGLT2is was shown to be independent of renal function, proving the existence of other factors beyond osmotic diuresis-dependent volume depletion [23]. Thus, direct vasodilation at the endothelial level mediated by NHE inhibition and decreased intracellular Ca^2+^ was proposed [24,25], as depicted in Figure 3. Furthermore, SGLT2is were shown to modulate the RAAS as well as the sympathetic nervous system [26,27], possibly contributing to reduce arterial stiffness [28].

Moreover, SGLT2is have been shown to have a direct vascular effect contributing to BP changes. Cooper et al. found that empagliflozin restored the integrity of the endothelial glycocalyx in human aortic cells, possibly resulting in an atheroprotective effect and contrasting endothelial dysfunction [29], which are two main features of diabetic and non-diabetic kidney injury. Furthermore, glycocalyx shedding [30] may trigger several abnormal pathways leading to a reduction in NO release [31] and impairment in renal sodium excretion [32,33,34].

The ability of SGLT2is to preserve and restore the structural integrity of the glycocalyx is remarkable, paving the way to maintaining vascular health by promoting a better sodium and BP balance, reducing oxidative stress and inflammation [35], and finally preventing the development of arterial stiffness [28] (Figure 3).

#### 2.2.2. Natriuresis and Fluid Volume Reduction

Volume reduction was demonstrated by SGLT2is even in the presence of GFR reduction below 45 mL/min and despite reduction in urine glucose excretion [36]. It was proposed that these drugs promote a negative sodium balance by preferentially mobilizing sodium from the interstitial compartment, thus preserving effective circulating volume and renal hemodynamics [37].

#### 2.2.3. Reduced Glucose and Lipo-Toxicity and Negative Caloric Balance

By driving glucose urinary excretion, SGLT2is induce negative glucose and caloric balance. Dapagliflozin was shown to attenuate gluco-toxicity, improve insulin sensitivity and plasma lipids profile as well as obesity-induced inflammation and oxidative stress [38], thus preventing renal fibrosis and leading to a reduction in body weight and visceral fat [39]. In addition, SGLT2is were shown to cause a shift in substrate utilization from glucose to FFAs, reducing the intracellular levels of toxic lipid metabolites, such as fatty acyl CoAs, diacylglycerol, and ceramides. These effects might prevent endoplasmic reticulum stress and pro-inflammatory and fibrotic processes as a result of oxidative stress reduction at the kidney level [40].

#### 2.2.4. Uric Acid

By increasing glucose concentration in the proximal tubule, where it competes with uric acid for the transporter GLUT9b, SGLT2is reduce reabsorption and promote uric acid urine excretion [26,41,42], probably contributing to reduce renal, cardiovascular and mortality risk [43,44,45]

In addition, it was suggested that SGLT2is may indirectly inhibit URAT1 through several mechanisms [46,47], including reduction in insulin secretion due to improvement in glucose metabolism [48]. However, serum UA changes induced by SGLT2is may be masked in CKD patients as glycosuria becomes smaller along with GFR reduction.

#### 2.2.5. Modulation of the Sympathetic Nervous and Renin Angiotensin Aldosterone Systems

SGLT2is were shown to increase urine volume and to reduce BP and weight without significant effect on SNS activity, in contrast with what is usually seen with other diuretic agents [49]. This suggested that the presence of factors induced by SGLT2is working to maintain SNS activity were unchanged despite BP reduction and volume depletion. Another possible explanation is that SGLT2-sympathetic inhibition was mediated by the central autonomic system, as suggested by the recent findings. By fact, SGLT2is inhibit central sympathetic as well as autonomic activity; however, the underlying mechanisms have not yet been clarified [50,51].

## 3. Renal Protection by SGLT2is: Data from CVOTs

From the publication of the Empagliflozin Cardiovascular Outcome Event Trial in Type 2 Diabetes Mellitus Patients (EMPA-REG OUTCOME trial) [52] in November 2015 to the more recent DAPA-CKD study [53], an impressive sequence of clinical trials has repeatedly confirmed the nephroprotective effect of SGLT2is, irrespective of blood-glucose-lowering effect [54].

In the first SGLT2is CVOT EMPA–REG OUTCOME [51,55], patients with type 2 diabetes at high-risk of CV were required to have an estimated glomerular filtration rate (eGFR) above 30 mL/min/1.73 m^2^; the mean eGFR was 74  ±  21 mL/min/1.73 m^2^ and 25.9% of patients had eGFR  < 60 mL/min/1.73 m^2^. As for albuminuria, 59.4% patients were normoalbuminuric, 29% microalbuminuric, and 11% macroalbuminuric. The composite renal outcome was incident or worsening nephropathy (i.e., progression to macroalbuminuria, doubling of serum creatinine level accompanied by an eGFR  ≤ 45 mL/min/1.73 m^2^, initiation of RRT, or renal death) and was lower in empagliflozin patients compared with placebo patients (HR  =  0.61, 95% CI 0.53–0.70, *p*  <  0.001). Notably, each component of the primary renal outcome was significantly reduced by the SGLT2is treatment. Patients on empagliflozin showed both a significantly lower risk of progression to macroalbuminuria (38% risk reduction) as well as less clinically relevant renal outcomes, such as a doubling of serum creatinine (44% risk reduction) and initiation of RRT (55% risk reduction), compared with those in the placebo group. The only insignificant difference was observed in the rate of incident albuminuria. Post hoc analyses showed that the use of empagliflozin is beneficial in terms of delaying renal disease progression independent of variation in albuminuria as well as baseline GFR.

Later on, in the CANagliflozin cardioVascular Assessment Study (CANVAS) program [26] patients were required to have eGFR above 30 mL/min/1.73 m^2^, the mean eGFR was 76.5  ±  20.5 mL/min/1.73 m^2^ and 20.1% of patients had eGFR  < 60 mL/min/1.73 m^2^. As for albuminuria, 69.8% of patients had normoalbuminuria, 22.6% microalbuminuria, and 7.6% macroalbuminuria. The composite renal outcome (i.e., 40% reduction in eGFR sustained for at least two consecutive measures, needed for RRT or renal death) rate was lower in patients treated with canagliflozin compared with the placebo group (HR  =  0.60, 95% CI 0.47–0.77, *p*  <  0.001). As for albuminuria, canagliflozin was associated with a 37% reduction in the rate of progression to macroalbuminuria and to a higher rate of regression to normoalbuminuria (HR 1.70, 95% CI 1.51–1.91).

In the Dapagliflozin Effect on Cardiovascular Events—Thrombolysis in Myocardial Infarction 58 (DECLARE-TIMI 58) study [56], patients were required to have creatinine clearance ≥60 mL/min with no specified minimum eGFR. Consequently, only 7.4% of patients had eGFR  < 60 mL/min. As for albuminuria, 67.9% patients had normoalbuminuria, 23.4% microalbuminuria, and 6.8% macroalbuminuria. The composite renal outcome (i.e., ≥ 40% reduction in eGFR to a threshold  <60 mL/min/1.73 m^2^, new end-stage renal disease or kidney transplantation, or renal/CV death) was lower in dapagliflozin patients compared with placebo patients (HR  =  0.76, 95% CI 0.67–0.87, *p*  <  0.001).

At variance with previous trials that have consistently shown renal protection through the use of SGLT2is, VERTIS CV (eValuation of ERTugliflozin effIcacy and Safety CardioVascular outcomes trial) [57] reported that the use of ertugliflozin was not associated with a significant risk reduction in renal composite outcomes (death from renal causes, RRT, or doubling of the serum creatinine level) even if trends for a beneficial effect on renal outcome were noted. However, subsequent analyses using similar renal endpoint definitions showed that both albuminuria reduction and GFR preservation over time are almost superimposable among different SGLT2is molecules and have a magnitude varying from 30 to 50% greater than the placebo [58].

These results led us to consider SGLT2is more for their potential to reduce the incidence of overt nephropathy than for their glucose-lowering effectiveness. Furthermore, the impact on nephroprotection appears to be reproducible and consistent in various clinical conditions and independent of GFR and albuminuria values.

## 4. Renal Protection by SGLT2is: Data from Renal Trials

The Canagliflozin and Renal Events in Diabetes with Established Nephropathy Clinical Evaluation (RCT CREDENCE) [26] was the first trial specifically designed to assess renal outcomes in a large cohort of 4401 patients with type 2 diabetes and chronic kidney disease (CKD). Study patients were required to have eGFR between 30 and 90 mL/min/1.73 m^2^ (in at least 60% patients with eGFR between 30 and 60 mL/min/1.73 m^2^) and to have macroalbuminuria. The study confirmed the beneficial effect of canagliflozin on the kidney in diabetic CKD patients and, of note, was prematurely terminated because of the evident benefit. Proteinuric patients with CKD stage 2 and 3 treated with canagliflozin showed significantly decreased risk of reaching the primary composite end-point of doubling of serum creatinine levels, end-stage kidney disease, or death for renal or cardiac causes over a median follow-up of 2.6 years (HR 0.70, 95% CI 0.59–0.82, *p* < 0.0001). Moreover, the benefits of canagliflozin seemed to be greater in patients with the worst kidney function and more severe proteinuria. Of the study patients, 15% had a documented history of heart failure at baseline and the secondary outcome of hospitalization for heart failure was significantly reduced (HR 0.61, 95% CI 0.47–0.80, *p* < 0.001).

More recently, the DAPA–CKD trial [53] showed a renal benefit in CKD patients independent of the presence of diabetes. In this trial CKD patients with (68%) or without (32%) diabetes with an eGFR 25 to 75 mL/min/1.73 m^2^ and a urinary albumin-to-creatinine ratio of 200 to 5000 mg/g were randomized to receive dapagliflozin or a placebo. The primary outcome was a composite of a sustained decline in the estimated GFR of at least 50%, end-stage renal disease, or death from renal or CV causes. Furthermore, as anticipated, the independent data monitoring committee recommended premature termination of the trial because of the efficacy demonstrated in the results. Over a median of 2.4 years, dapagliflozin significantly reduced the primary outcome event (HR 0.61; 95% CI 0.51 to 0.72; *p* < 0.001). Furthermore, dapagliflozin significantly reduced the risk of composite renal events (HR 0.56, 95% CI 0.45–0.68; *p* < 0.001) as well as the risk of the composite of CV death or hospitalization for heart failure (HR 0.71, 95% CI 0.55–0.92; *p* = 0.009). Notably, this benefit was enjoyed both by patients with diabetes (36% risk reduction) and, even more, by patients without diabetes (50% risk reduction). Therefore, the DAPA-CKD study contributes to the knowledge obtained from the CREDENCE trial, extending the nephroprotective effect of SGLT2is to CKD patients without diabetes.

### Ongoing Studies

The ongoing EMPA-KIDNEY trial [59] is expected to confirm and extend preliminary data from DAPA-CKD by investigating the role of empagliflozin in a cohort of over 6000 CKD patients, mostly non-diabetic and with relatively low albuminuria levels. The results of this trial, expected before the summer of 2022, will inform clinicians on the potential application of SGLT2is to a broader phenotype of CKD patients, very often encountered in real-world clinical practice.

## 5. Conclusions and Perspectives

In recent years, following the publication of CVOTs and later RCTs focused on nephroprotection, exciting emerging results on renal and CV benefits with the use of SGLT2is were promptly acknowledged and incorporated into international guidelines for treatment and prevention of DKD. In 2018, both the American Diabetes Association and the European Association for the Study of Diabetes published a consensus statement on the management of hyperglycemia in patients with type 2 diabetes, emphasizing the importance of exploiting the organ protection features of specific antihyperglycemic drugs in the management of type 2 diabetes [60]. SGLT2is are now recommended as a part of glucose-lowering regimens among patients with established atherosclerotic cardiovascular diseases, kidney disease, multiple atherosclerotic cardiovascular disease risk factors, or DKD (if eGFR is adequate) to reduce the risk of major adverse cardiovascular events and heart-failure-related hospitalization. If ongoing studies confirm that the benefits previously observed in diabetic patients can also be observed in the broader, non-diabetic population, this class of drugs could soon become the standard of care for the whole population of patients at renal risk.

## Data Availability

Not applicable.
